# Adaptive SIR model with vaccination: simultaneous identification of rates and functions illustrated with COVID-19

**DOI:** 10.1038/s41598-022-20276-7

**Published:** 2022-09-20

**Authors:** Tchavdar T. Marinov, Rossitza S. Marinova

**Affiliations:** 1 Department of Natural Sciences, Southern University at New Orleans, 6801 Press Drive, New Orleans, LA 70126 USA; 2 Department of Mathematical and Physical Sciences, Concordia University of Edmonton, 7128 Ada Boulevard, Edmonton, AB T5B 4E4 Canada; 3 Department of Computer Science, Varna Free University, Varna, Bulgaria

**Keywords:** Biophysics, Computational biology and bioinformatics, Mathematics and computing

## Abstract

An Adaptive Susceptible-Infected-Removed-Vaccinated (A-SIRV) epidemic model with time-dependent transmission and removal rates is constructed for investigating the dynamics of an epidemic disease such as the COVID-19 pandemic. Real data of COVID-19 spread is used for the simultaneous identification of the unknown time-dependent rates and functions participating in the A-SIRV system. The inverse problem is formulated and solved numerically using the Method of Variational Imbedding, which reduces the inverse problem to a problem for minimizing a properly constructed functional for obtaining the sought values. To illustrate and validate the proposed solution approach, the present study used available public data for several countries with diverse population and vaccination dynamics—the World, Israel, The United States of America, and Japan.

## Introduction

Infectious diseases modelling attracted great deal of attention by scientists and people around the world during the COVID-19 pandemic. The novel coronavirus (SARS-CoV-2) started to quickly spread in the early months of 2020 and was announced as a global pandemic by the World Health Organization^1^ in March 2020. Population-wide vaccination is critical for achieving herd immunity and for controlling the COVID-19 pandemic while combined with effective testing and preventive measures.

Inevitably, the development of vaccines became the highest priority of governments and pharmaceutical companies^2^. Several vaccines were available in the last months of 2020. As of 30 July 2021, over 28% of the world population is partly or fully vaccinated^3^. Not knowing much about the coronavirus disease in the early months, mathematical models have played an important role in shedding some light on the disease dynamics.

The SIR model categorizes individuals as Susceptible, Infectious, and Recovered. Mathematical infectious disease models based on the classical SIR model^4^ are widely used to examine the spread of a disease. These models display compelling results especially during the early period of the pandemic^5–19^. In a recent article^20^, the authors studied the immune response of recovered from COVID-19 individuals up to 8 months patients and found that they have considerable immune memory.

Vaccination is a common method of reducing infectious diseases spread^21–25^ because it reduces the number of susceptible, from where the reproduction number naturally also decreases. The reproductive number is an indicator of the transmissibility of the virus caused by infectious individuals. It is affected by the population density, vaccinations, quarantines, social distancing, mask wearing and other measures^25,26^.

In a recent work^27^, the authors present a study of the temporal evolution of epidemic outbreaks accounting for vaccinations with monitored real time COVID-19 data, using the SIRV model, V denoting the relative fractions of currently vaccinated. They make certain assumptions and reduce the time-dependent general SIRV equations to an analytical model. SIR and SEIR models with vaccination are used to simulate and predict the development of the COVID-19 spread, e.g.^25,28–30^.

In several very recent publications^31–35^ applied to the COVID-19 epidemic, researchers have developed and used SIR and SEIR based models with vaccination to overcome the limitations of the conventional SIR model. The work in^31^ presents an investigation of the dynamics of a stochastic SIRV epidemic model with general non-linear incidence and vaccination. The introduced random fluctuations controls the disease outbreak. Zhao et al.^32^ use improved SIRV to evaluate the performance of non-pharmaceutical interventions in reducing the number of daily new cases of COVID-19 in South and Southeast Asia. They apply statistical methods to estimate parameters.

The research presented in^33^ proposes an SIRV evolutionary game model for infectious disease vaccination strategies based on the scale-free networks with tunable clustering. Their model analyzes the vaccination strategies taking into account factors such as vaccination effectiveness, vaccination cost, treatment cost after illness, government subsidy rate and treatment discount rate. Other notable work is about modelling infectious diseases with herd immunity in a randomly mixed population^34^. The authors formulate two new SIR models to mimic the declining transmission rate of infectious diseases at different stages of transmission. They found that natural herd immunity might not be sufficiently effective in infectious diseases with high reproduction numbers.

Researchers implemented a modification of a SIR model^35^ to study the role of the rate of vaccination, rate of transmission and the likelihood of emergence of resistant strains. They used parameters realistically resembling SARS-CoV-2 transmission to run simulations for a total time of three years, with vaccination starting one year into the model.

The goal of the present work is to develop and to demonstrate the effectiveness of an inverse method for identifying the time-dependent functions and parameters of the SIRV model simultaneously (Sect. 2). We apply the method to the adaptive SIRV (A-SIRV) epidemic model using publicly available COVID-19 data. In contrast to other works which use statistical approaches to estimate parameters, we apply an inverse problem approach to identify these parameters. The dynamically estimated rates can be particularly useful in running other simulations, such as in^35^, to study the epidemic.

## The modified SIR model for the spread of an infectious disease and vaccination (SIRV)

The standard notations in the SIR model are: *S*(*t*) denotes the number of *susceptible*, *I*(*t*) – *infectives*, and *R*(*t*) – *removed* individuals. Assume that the time-dependent function $u=u(t)$ represents the *vaccination rate*. Then the total number of vaccinated individuals is given by

1 $$\begin{aligned} V(t)=\int _0^t u(t)dt. \end{aligned}$$

Every vaccine has different level of efficacy. We assume that the vaccine efficacy impacts disease spread and prevents transmission at the same rate, which is a reasonable assumption according to the study^35^. If people are vaccinated with the same vaccine type / brand, it is possible to introduce the efficacy of the vaccine in the model. Since there is lack of information about the vaccine types and other details concerning the vaccinated individuals, we make the following assumptions, which may not be true for all

- Individuals being vaccinated belong to the class *S* (susceptible) before the vaccine. There are recovered people who vaccinate themselves; this is not included in the model because of lack of data.
- Individuals move to the class *R* (removed) after vaccination. In other words, the model assumes the vaccine is 100% effective against the disease, namely vaccinated people become fully immune.

Due to unavailability of data about COVID-19 variants and vaccine details on a country level, at this stage, the model does not include important assumptions, such as:

- Vaccinated individuals could be infected and be infectious if the infection is caused by other variants, known as the vaccine breakthrough problem; there might be multiple variants circulated in the same country, not only a single strain.
- Infected individuals could be vaccinated again to improve their immune level; vaccinated individuals could be infected again due to waning of immunity for COVID-19.

**Figure 1:**
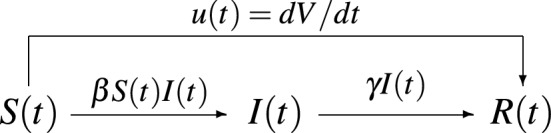
The A-SIRV epidemic model.

The *transmission rate* $\beta >0$ gives the probability that a random infective person infects a random susceptible person. A major approximation here is the assumption that the population under study is well mixed so that every person has equal probability of coming into contact with every other person. The *removal rate* $\gamma >0$ gives the probability that an infective person recovers. In the classical SIR model $\beta$ and $\gamma$ are constants, while they are functions of time in^14^. The diagram in Fig. 1 describes the adaptive SIR (A-SIR) model with vaccinations (A-SIRV) and time-dependent coefficients:

2 $$\begin{aligned} L_1 (S,I,\beta )&=\frac{dS(t)}{dt} +\beta (t) S(t)I(t) + u(t) = 0 \end{aligned}$$

3 $$\begin{aligned} L_2 (S,I,\beta , \gamma )&= \frac{dI(t)}{dt} - \beta (t) S(t)I(t) +\gamma (t) I(t) = 0 \end{aligned}$$

4 $$\begin{aligned} L_3 (I,R, \gamma )&=\frac{dR(t)}{dt} - \gamma (t) I(t) - u(t) = 0. \end{aligned}$$

The total population $N = S(t)+I(t)+R(t)$ is considered constant in the equations. Fig. 1 shows the diagram for the A-SIRV model, corresponding to the system (2)–(4).

The A-SIRV model used in this study assumes that the removed individuals are no longer susceptible nor infectious. The number of cases for recovered from COVID-19 individuals who are re-infected at the present moment is very limited and the rate cannot be estimated; thus, this possibility in not taken into account.

### Effective and basic reproductive ratios $R_e$ and $R_0$

An epidemic occurs if an infective individual introduced into a population of susceptible individuals infects on average more than one other person, namely *I*(*t*) is increasing in time. The original SIR model assumes that the transmission and removal rates are constants. Equations (2)–(4), with proper initial conditions, allow the determination of *I*(*t*), and *S*(*t*), and *R*(*t*), if the coefficients $\beta$ and $\gamma$ are known constants. However, in the case of a pandemic, the rates may vary in time; hence, $\beta =\beta (t)$ and $\gamma =\gamma (t)$.

The so-called *effective reproduction number* (also *effective reproduction rate* or *ratio*) for a given epidemic is the parameter $R_e(t)$ given by

5 $$\begin{aligned} R_e(t)=\frac{\beta (t) S(t)}{\gamma (t)}. \end{aligned}$$

An epidemic occurs when $R_e(t)>1$. Then the fraction of the population that is immune increases (because of vaccination or because of recovering from the disease) so much that $R_e(t)<1$, *”herd immunity”* is achieved. Hence, the number of new cases occurring in the population will decrease to zero.

Another important characteristic of an epidemic, *the basic reproduction number (or ratio, or rate)* $R_0$, is defined as:

6 $$\begin{aligned} R_0=\frac{\beta (t) N}{\gamma (t)}, \end{aligned}$$

where *N* is the size of the total population.

Multiple factors may cause the rates to change over time. In the case of COVID-19, examples include social distancing, restrictions imposed by governments, and preventive treatments. Therefore, we define the effective reproduction number and the basic reproduction number to be functions of time, defined in (5) and (6), respectively.

The main goal of the present work is to identify the time-dependent reproduction rates directly using the SIRV model.

### Inverse problem formulation

The initial-value problem consisting of the system of equations (2)–(4), with coefficients $\beta (t)$ and $\gamma (t)$ known, along with proper initial conditions derived from the given data, constitutes the direct problem. Note that the vaccination rate function *u*(*t*) in equations (2) and (4) can be obtained from the function *V*(*t*), which is known from the reported public data on a daily basis.

In reality, the values of the time-dependent parameters $\beta (t)$ and $\gamma (t)$ are unknown for a new epidemic disease. Hence, the simultaneous determination of the coefficients and functions from the available data is an *inverse problem*.

**Figure 2:**
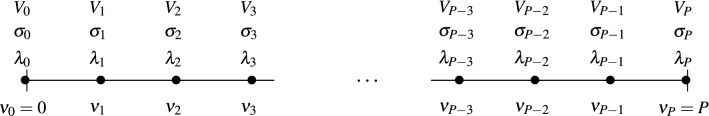
The time nodes $\nu _i$ where the data values are given.

Let [0, *P*], where *P* is a number of days, be a time sub-interval (Fig. 2), where approximate values of the susceptible *S*(*t*) and currently infectious *I*(*t*) are known at specified time moments $\nu _0, \nu _1, \dots , \nu _P$,

7 $$\begin{aligned} S(\nu _i)\approx \sigma _i, \qquad I(\nu _i) \approx \lambda _i,\qquad i=0,1,\dots , P. \end{aligned}$$

At the same time, the values of $V(\nu _i)$ are given exactly since the number of vaccinated people is available, as reported in^3^. Therefore, we can approximate the data values of *V*(*t*) using a function with a continuous second derivative. From this function, the values $u(t)=\frac{d V}{d t}$ can be estimated.

Since the values of the vaccination rate function *u*(*t*) can be found from the available data of vaccinated individuals, we can find *I*(*t*), *S*(*t*), $\beta (t)$, and $\gamma (t)$ from equations (2), (3), and (7). Note that this problem is inverse and require a special treatment. The approach used here is based on the Method of Variational Imbedding (MVI)^14,36^. Following the idea of MVI, we construct a functional using the original equations (2), (3), and the available data values (7). We define the functional over the sub-interval [0, *P*] as

8 $$\begin{aligned} {\mathscr {F}}= \int _0^P \left[ L_1^2+L_2^2 +\sum _{i=1}^{P-1} \delta (t-\nu _i) \ \mu _i \left( (S(t)-\sigma _{i})^2+(I(t)-\lambda _{i})^2 \right) \right] d t, \end{aligned}$$

where $\mu _i$ are the weights prescribed for the *i*-th node and $\delta (t)$ is the *Dirac delta function* $\delta (t-\nu _i)$ defined as:

$$\begin{aligned} \delta (t-\nu _i) = {\left\{ \begin{array}{ll} \infty , &{} t = \nu _i \\ 0, &{} t \ne \nu _i \end{array}\right. } \quad \text { and } \quad \int _{-\infty }^{\infty } \delta (t-\nu _i) d t = 1. \end{aligned}$$

In other words, we substitute the problem for finding the unknown functions *I*(*t*), *S*(*t*) and the coefficients $\beta (t)$, $\gamma (t)$ in equations (2), (3), and (7) with a problem for minimization of the functional ${\mathscr {F}}$ defined by (8). Finding *R*(*t*) is straightforward after knowing the values of *I*(*t*), *S*(*t*), $\beta (t)$, and $\gamma (t)$.

The absolute minimum of ${\mathscr {F}}$ is equal to zero with the functional becoming zero if and only if equations (2), (3) and conditions (7) are satisfied. We want to emphasize here that the functions (*S*, *I*, *V*) and the parameters $(\beta ,\gamma , u)$ are unknown and one have to identify them simultaneously by solving a minimization problem.

## Method for solving the inverse problem with time-dependent rates

In order to solve the inverse problem with time-dependent rates, we first find the minimum of the functional ${\mathscr {F}}$, defined in (8), numerically over a sub-interval of the entire period assuming constant transmission and recovery rates. The details of solving the minimization sub-problem are given in “Appendix A”.

**Figure 3:**
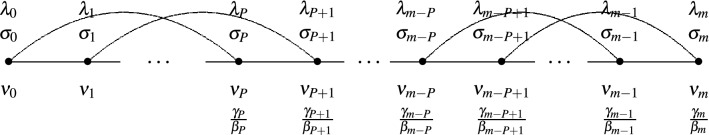
The time nodes and the subsets of fixed length of $P+1$ days for identifying $\beta (t)$ and $\gamma (t)$.

Let the values of *S*(*t*) and *I*(*t*) be known at some time moments $\nu _0,\nu _1,\dots ,\nu _m$, shown in Fig. 3, namely

9 $$\begin{aligned} S(\nu _l)=\sigma _l, \quad I(\nu _l)=\lambda _l, \quad \text { for }\quad l=0,1,\dots , m. \end{aligned}$$

We consider the following two approaches for estimating the coefficients $\beta _l$ and $\gamma _l$ in the system of equations (2), (3):

i. Solve the inverse problem for the system of equations (2), (3) under the boundary conditions
  10 $$\begin{aligned} S(\nu _{l-1})=\sigma _{l-1}, \quad I(\nu _{l-1})=\lambda _{l-1}, \quad S(\nu _l)=\sigma _l, \quad I(\nu _l)=\lambda _l, \end{aligned}$$
  for $l=1,2,\dots , m$. This approach is similar to the method developed in^37^ for estimating the coefficient in Euler-Bernoulli equation, later modified in^36^ for the SIR equations. It works well if the data represent the exact values of the functions *S*(*t*) and *I*(*t*). In a real-world data, e.g. the available public data for the COVID-19 pandemic, there usually exists random noise causing oscillations in the numerical results.
ii. Use the solution method, proposed in Sect. 3, for estimating the parameters $\beta$ and $\gamma$ as constants on every sub-interval $[\nu _l, \nu _{l+P}]$, Then, we use the obtained constant values to approximate the non-constant values $\beta _{l+P}$ and $\gamma _{l+P}$ for $l=0,1,\ldots ,m-P$. This approach is smoothing the data automatically. Knowing the approximate values of the transmission and recovery rates, we obtain the reproduction rates
  $$\begin{aligned} R_{0,k} = \frac{\beta _k}{\gamma _k} N \quad \text {and} \quad R_{e,k} = \frac{\beta _k}{\gamma _k} \sigma _l, \qquad k=P,P+1,\ldots ,m. \end{aligned}$$

## Results

The numerical simulations have been performed using the available public data from^3^ and^38^ websites. The number of currently infected persons, $\lambda _k$, is reported daily on^38^. Both websites^3^ and^38^, report the total number of infected individuals, $T_k= I_k+R_k$ from the beginning of the COVID-19 pandemic.

The daily number of vaccinated people, $V_k$, is taken from^3^. We assume that the reported data is correct. In order to to obtain a smooth approximation of the vaccinated persons *V*(*t*) and the vaccination rate $u(t)=dV/dt$, we use cubic spline approximation based on the values $V_k$. Consequently, the number of susceptible individuals, $\sigma _k$, for a given country can be found in the following way: $\sigma _k=N-T_k-V_k$, where *N* is the total population.

We do not pretend that the available data are accurate. For sure, the so-called ”hidden cases” (asymptotic cases or cases without official tests) are not included in the reported data. Moreover, the posted data contain random (human) mistakes which can be treated as random noise. Here, we use the posted data to illustrate the method described in "Method for solving the inverse problem with time-dependent rates" section.

The presented results for the rates $N \beta$, $\gamma (t)$, $R_0(t)$, and $R_e(t)$ are based on 28-day, 35-day, and 42-day sub-periods, over the entire multi-month period, from August 2020 until August 3, 2021.

The selected countries (Israel, United States, and Japan) for this study represent population with different vaccination dynamics. Israel performed early aggressive vaccination during the first half of 2021, United States is catching up, whereas Japan is behind compared to them. The World is aa good example of aggregated global data. Table 1.

**Table 1:** Vaccinated, recovered, and susceptible for the World and selected countries as of August 3, 2021.

	Fully vaccinated (%)	Recovered (%)	Susceptible (%)
World	14.78	2.56	82.66
Israel	57.70	9.43	32.87
United States	49.54	10.79	39.67
Japan	30.10	0.75	69.15

### The World

**Figure 4:**
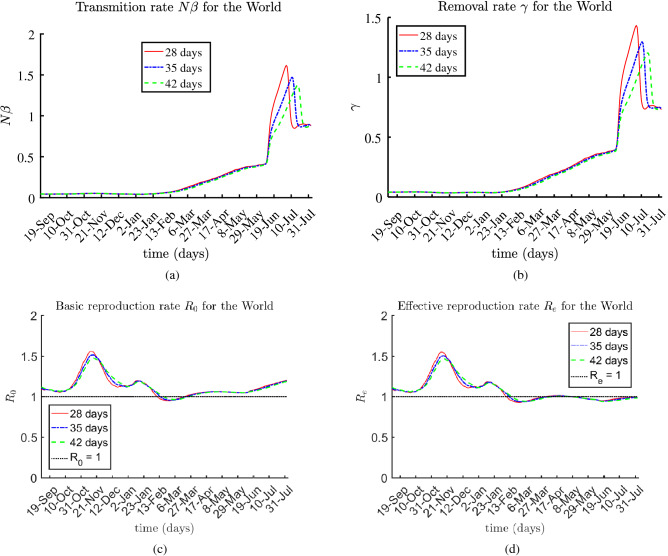
Estimated rates $N \beta$, $\gamma$, $R_0$, and $R_e$ for the World.

 Estimated rates $N \beta$, $\gamma$, $R_0$, and $R_e$ for the World are shown at Fig. 4. According to the reported data as of August 3, 2021: $14.78\%$ of the population was fully vaccinated; about $2.56\%$ of the population met the virus. Thus, $82.66\%$ of the population remained susceptible. The basis and effective reproduction rates have been near 1 since January 2021.

### Israel

**Figure 5:**
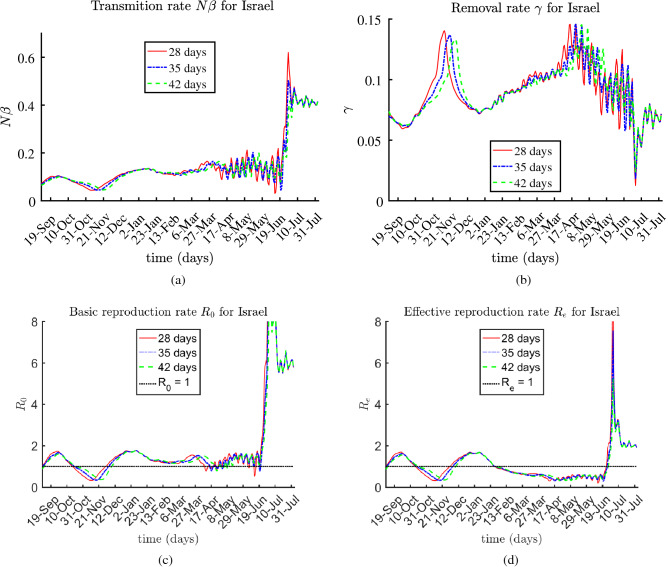
Estimated rates $\beta N$, $\gamma$, $R_0$, and $R_e$ for Israel.

During the first half of 2021, Israel was the COVID-19 vaccination champion – over $57.7\%$ of the population of the country was vaccinated by the end of July. At the same time, according to the reports, approximately $9.43\%$ of the population met the virus; hence, only $32.87\%$ of the population was susceptible. According to^21^, early mass vaccination programs predict a reduction of the effective reproduction rate of infection within communities. The estimated values of the basic and effective reproduction ratios $R_0$ and $R_e$ for Israel are shown in Fig. 5. The effective reproduction rate is practically constant, slightly less then 1 from January to middle June 2021. In June 2021, the rates increased significantly for a short period of time. Then, the reproduction rates decreased but they still remained above 1.

### The United States of America

**Figure 6:**
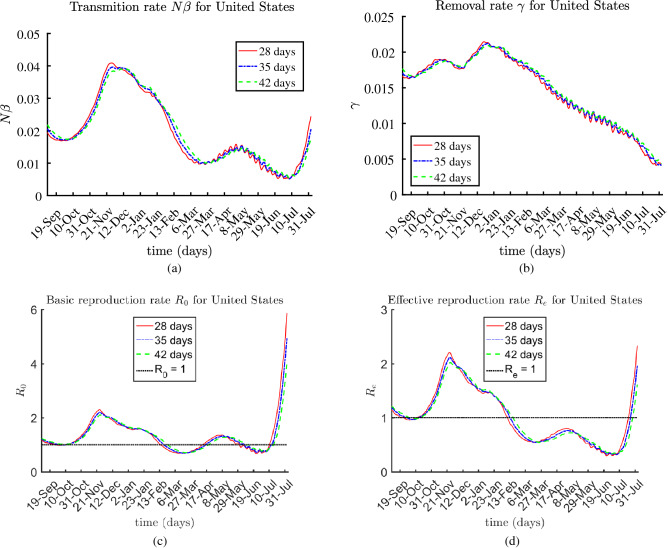
Estimated rates $N \beta$, $\gamma$, $R_0$, and $R_e$ for United States.

 The reported data for The United States of America state: $49.54\%$ were fully vaccinated, about $10.79\%$ of the population met the virus; hence, $39.67\%$ of the population were susceptible. The estimated values of the transmission rate $N\beta$, $\gamma$, the basic and effective reproduction ratios $R_0$ and $R_e$ are shown at Fig. 6. The transmission rate was the highest in fall 2020. It started to increase again in June-July 2021. COVID-19 is on the rise in many countries, casing a new wave. This surge is due to widespread resumption of normal activities.

### Japan

**Figure 7:**
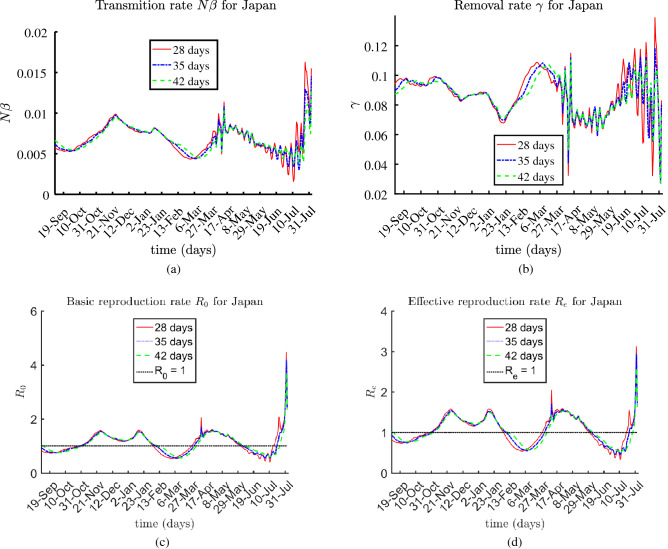
Estimated rates $\beta N$, $\gamma$, $R_0$, and $R_e$ for Japan.

 Japan is with about $30.10\%$ fully vaccinated individuals, approximately $0.75\%$ reported to be totally infected; this, about $69.15\%$ are susceptible;. The estimated values of the transmission rate $N\beta$, $\gamma$, the basic and effective reproduction rates $R_0$ and $R_e$ are shown in Fig. 7. While the rates had been relatively reasonable and low until recently, they started to increase lately. This growth can be explained with the 2020 Summer Olympics held from 23 July to 8 August 2021 in Japan.

## Discussion

We performed numerical simulations with the developed method for the A-SIRV model for identifying the transmission, removal and reproduction rates globally and for three countries (Israel, United States, and Japan) using publicly available data. Estimated rates for the World show the presence of three waves and a forth wave being formed since July 2021.

The values for the identified rates for the World do not oscillate, regardless of the number of days *P*. By contrast, the obtained rates of the selected individual countries have fluctuations during 2021, which are smaller for $P=42$ compared to $P=28$ and $P=35$. This is because a larger time period is having a smoothing effect of the oscillating functions.

We observe oscillations during time periods that include vaccination data. The vaccination rate *u*(*t*) is approximated from the given data for the number of vaccinated individuals *V*(*t*). It clearly affects the computed rates in the A-SIRV equations. The oscillations in the rates for Israel, Japan, and to some extent United States can be explained with fluctuations in the vaccination rate *u*(*t*) due to irregularities in the data for vaccinated people *V*(*t*). We assume that the problem is posed correctly, namely the data have ”physical meaning” and, therefore, a solution of the problem exists, see^39^. The randomness of the vaccination data introduces oscillations in the obtained numerical values of the rates.

Finally, it is important to mention that the A-SIRV model considers only one homogeneous population for the selected countries: thus, giving aggregated results for the estimated rates and the unknown functions. The populations of The World and The United State of America are obviously not homogeneous. Such results can still help see trends of potential future growth of the epidemics and, if needed, guide the design of alternative interventions.

## Conclusions

Mathematical models can help with visualizing and predicting the long-term behaviour of an infectious disease, despite of the fact that there are many limitations to using them. For instance, the SIR type models contain many assumptions such as: accuracy and completeness of reported data; mixing of the population; no reinfection; constant population; and so forth. The present work studies the performance of a method for an epidemic based on an inverse problem approach for estimating the time-dependent transmission and removal rates in the A-SIRV epidemic model. The inverse problem is solved by defining a minimization problem using the entire dataset for the examined population, with available COVID-19 data. The work utilizes an inverse problem approach to the time-dependent transmission and removal rates identification as well as the unknown functions in the A-SIRV (Adaptive SIRV) model. This can give insight into how well the method identifies the parameters that can be used to predict the infectious disease spread. If conditions change, then the predictions may no longer be accurate; hence, adjustments will be required based on the existing conditions, for obtaining new predictions.

## Data Availability

The COVID-19 data is publicly available at https://ourworldindata.org/coronavirus-source-data and https://www.worldometers.info/coronavirus/.

## Appendix A: Solving the minimization sub-problem

In order to solve the minimization sub-problem used in Sect. 3, we first approximate the derivatives and integrals in (8).

### A.1 Discretization of the minimization sub-problem

Let $\tau = \frac{P}{n}$ be the time-step of a uniform grid on the finite interval [0, *P*], where *n* is the total number of grid nodes. The grid nodes are defined as: $t_k=k\tau$, $k=0,1,\dots ,n$. It is important that $\tau$ (respectively *n*) is chosen to ensure that every time moment $\nu _1,\nu _2,\dots , \nu _P$ coincides with one grid node. Let $S_k$, $I_k$, and $u_k$ be notations for the corresponding grid values of the functions $S(t_k)$, $I(t_k)$, and $u(t_k)$.

Since the problem for minimization of the functional ${{\mathscr {F}}}$ is non-linear, it requires lineariation at some stage of the solution process. Let $\hat{S_k}$, $\hat{I_k}$ be known approximate values (say from the previous iteration), used in the non-linear term of the differential equations (2), (3). Such linearization has the advantage that it allows building an iterative procedure with a time-independent matrix of the resulting linear system. Hence, we invert this matrix once, at the start of the iterations.

In order to secure $O(\tau ^2)$ errors of approximation of the operators in (8), we discretize the derivatives in $L_1$ and $L_2$ at the grid nodes $t_{k-1/2}$. We introduce the notations for the approximations of *S*(*t*) and *I*(*t*) at the midpoints of $[t_{k-1}, t_{k}]$, where $k=1,2,\ldots , n$:

$$\begin{aligned} {\hat{S}}_{k-1/2} = 0.5({\hat{S}}_{k-1}+{\hat{S}}_k), \quad {\hat{I}}_{k-1/2} = 0.5({\hat{I}}_{k-1}+{\hat{I}}_k), \quad u_{k-1/2} = 0.5(u_{k-1}+u_k). \end{aligned}$$

Consequently, the differential operators $L_1$ and $L_2$ are approximated by the following linear difference operators $\Lambda _{1}$ and $\Lambda _{2}$

11 $$\begin{aligned} \Lambda _{1,k}&=\frac{S_k-S_{k-1}}{\tau } +\beta {\hat{S}}_{k-1/2}{\hat{I}}_{k-1/2}+ u_{k-1/2} \end{aligned}$$

12 $$\begin{aligned} \Lambda _{2,k}&= \frac{I_k-I_{k-1}}{\tau } - \beta {\hat{S}}_{k-1/2}{\hat{I}}_{k-1/2} +\gamma {\hat{I}}_{k-1/2}, \end{aligned}$$

for $$k=1,2,\ldots , n$$.

We approximate $\delta (t_k-\nu _i)$ as

13 $$\begin{aligned} \delta (t_k-\nu _i) = {\left\{ \begin{array}{ll} \dfrac{1}{\tau }, &{} \quad t_k = \nu _i \\ 0, &{} \quad t_k\ne \nu _i \end{array}\right. }\quad \text { for }\quad k=1,2,\ldots , n,\quad i=1,2,\ldots , P-1. \end{aligned}$$

As already stated, it is important that for every $1\le i\le P-1$, there exists index $k_i$, such that $\nu _i=t_{k_i}$, i.e., the set of time moments $\{\nu _0,\nu _1,\dots , \nu _P\}$, is a subset of the set of grid nodes $\{t_0, t_1, \dots , t_n\}$. To simplify the presentation, let us introduce the notations

$$\begin{aligned} \bar{\mu _k} = {\left\{ \begin{array}{ll} \mu _i, &{} t_k=\nu _i, \\ 0, &{} \text {otherwise} \end{array}\right. } \quad \bar{\sigma }_k = {\left\{ \begin{array}{ll} \sigma _i, &{} t_k=\nu _i, \\ 0, &{} \text {otherwise} \end{array}\right. } \quad \bar{\lambda }_k = {\left\{ \begin{array}{ll} \lambda _i, &{} t_k=\nu _i, \\ 0, &{} \text {otherwise} \end{array}\right. } . \end{aligned}$$

In other words, the values $\bar{\mu }$, $\bar{\sigma }$, and $\bar{\lambda }$ on the grid $\{t_0, t_1, t_2,\dots , t_n\}$ are equal to $\mu$, $\sigma$, and $\lambda$, respectively, if there exists $k\in \{0,1,\dots ,n \}$ such that $t_k=\nu _i$, $1\le i\le P-1$. Otherwise, for $t_k\ne \nu _i$ the values $\bar{\mu }$, $\bar{\sigma }$, and $\bar{\lambda }$ are zero.

This way, the discretized version of the functional ${\mathscr {F}}$ becomes

14 $$\begin{aligned} \Phi = \sum _{k=1}^n \left[ (\Lambda _{1,k}^2 + \Lambda _{2,k}^2)\tau + \bar{\mu }_k \left( (S_{k}-\bar{\sigma }_k)^2 +(I_{k}-\bar{\lambda }_k)^2 \right) \right] . \end{aligned}$$

### A.2 Equations for *S* and *I*

The necessary conditions for minimization of the function $\Phi$ with respect to its arguments $S_k$ and $I_k$ are

15 $$\begin{aligned} \frac{\partial \Phi }{\partial S_k}=0, \qquad \frac{\partial \Phi }{\partial I_k}=0. \end{aligned}$$

For the case under consideration, we obtain the following equations in the internal grid nodes $t_k$, where $k=1,2,\dots ,n-1$

16 $$\begin{aligned}&S_{k-1}-(2+\tau \mu _k)S_k +S_{k+1} \nonumber \\&= -\tau \beta ({{\hat{S}}}_{k+1/2} {{\hat{I}}}_{k+1/2} - {{\hat{S}}}_{k-1/2} {{\hat{I}}}_{k-1/2})-\tau (u_{k+1/2}-u_{k-1/2})-\tau \mu _k\sigma _k, \end{aligned}$$

17 $$\begin{aligned}&I_{k-1}-(2+\tau \mu _k)I_k +I_{k+1} \nonumber \\&= \tau \beta ({{\hat{S}}}_{k+1/2} {{\hat{I}}}_{k+1/2} - {{\hat{S}}}_{k-1/2} {{\hat{I}}}_{k-1/2}) -\tau \gamma ({{\hat{I}}}_{k+1/2} -{{\hat{I}}}_{k-1/2} ) -\tau \mu _k\lambda _k. \end{aligned}$$

The equations at the start grid node $t_0$ and the end grid node $t_n$ are

18 $$\begin{aligned} S_1-S_{0}&= \tau (-\beta {\hat{S}}_{1/2}{\hat{I}}_{1/2}- u_{1/2} ), \end{aligned}$$

19 $$\begin{aligned} I_1-I_{0}&= \tau ( \beta {\hat{S}}_{1/2}{\hat{I}}_{1/2} -\gamma {\hat{I}}_{1/2} ), \end{aligned}$$

20 $$\begin{aligned} S_n-S_{n-1}&= \tau (-\beta {\hat{S}}_{n-1/2}{\hat{I}}_{n-1/2}- u_{n-1/2} ), \end{aligned}$$

21 $$\begin{aligned} I_n-I_{n-1}&= \tau ( \beta {\hat{S}}_{n-1/2}{\hat{I}}_{n-1/2} -\gamma {\hat{I}}_{n-1/2} ), \end{aligned}$$

This way, given that approximate values of $\beta$ and $\gamma$ are known from the previous iteration, there are two well-posed systems of $(n+1)$ linear equations: (16), (18), and (20) for the unknown set of new values $(S_0, S_1, S_2, \dots , S_n)$; and (17), (19), and (21) for $(I_0, I_1, I_2, \dots , I_n)$. Due to the early linearization, these two systems are with constant matrices. Hence, we need to invert them once and use the inverse matrices during the entire iterative process.

### A.3 Equations for $\beta$ and $\gamma$

We rewrite the function $\Phi$ in the form

22 $$\begin{aligned} \Phi =\alpha _{00} +\alpha _{10} \beta +\alpha _{01}\gamma +\alpha _{20}\beta ^2 +\alpha _{11}\beta \gamma +\alpha _{02}\gamma ^2, \end{aligned}$$

where

$$\begin{aligned} \alpha _{00}= & {} \sum _{k=1}^{n} \left[ (I_k-I_{k-1})^2+(S_k-S_{k-1})^2 +\tau ^2 u_{k-1/2}^2 \right] ,\\&+ \bar{\mu }_k \left[ (S_{k}-\bar{\sigma }_k)^2 +(I_{k}-\bar{\lambda }_k)^2 \right] ,\\ \alpha _{10}= & {} \sum _{k=1}^{n} -2 {{\hat{I}}}_{k-1/2} {{\hat{S}}}_{k-1/2} (I_k - I_{k-1} - S_k + S_{k-1} -\tau u_{k-1/2}) \tau , \\ \alpha _{01}= & {} \sum _{k=1}^{n} 2 {{\hat{I}}}_{k-1/2} (I_k - I_{k-1}) \tau , \\ \alpha _{20}= & {} \sum _{k=1}^{n} 2 {{\hat{I}}}_{k-1/2}^2 {{\hat{S}}}_{k-1/2}^2 \tau ^2, \, \alpha _{11}=\sum _{k=1}^{n} -2 {{\hat{I}}}_{k-1/2}^2 {{\hat{S}}}_{k-1/2} \tau ^2, \, \alpha _{02}=\sum _{k=1}^{n} {{\hat{I}}}_{k-1/2}^2 \tau ^2. \end{aligned}$$

The necessary conditions for minimization of the function $\Phi$ with respect to $\beta$ and $\gamma$ are given by

23 $$\begin{aligned} \frac{\partial \Phi }{\partial \beta } =&\alpha _{10} + 2 \alpha _{20} \beta + \alpha _{11} \gamma =0, \end{aligned}$$

24 $$\begin{aligned} \frac{\partial \Phi }{\partial \gamma } =&\alpha _{01} + \alpha _{11} \beta + 2\alpha _{02} \gamma =0. \end{aligned}$$

The solution of the system (23), (24) is

25 $$\begin{aligned} \beta =&-\frac{2 \alpha _{02} \alpha _{10} - \alpha _{01} \alpha _{11}}{-\alpha _{11}^2 + 4 \alpha _{02} \alpha _{20} } , \end{aligned}$$

26 $$\begin{aligned} \gamma =&-\frac{\alpha _{10} \alpha _{11} - 2 \alpha _{01} \alpha _{20}}{\alpha _{11}^2 - 4 \alpha _{02} \alpha _{20}} . \end{aligned}$$

Up to this point, we have constructed two systems of linear equations, one system for (*S*, *I*) under the condition that $(\beta ,\gamma )$ are given, and another system for $(\beta ,\gamma )$ under the condition that (*S*, *I*) are given. This allows us to build an algorithm for finding a solution to the entire non-linear problem, by means of an iterative procedure, replacing $(\beta , \gamma )$ (when calculating (*S*, *I*)), or (*S*, *I*) (when calculating $(\beta , \gamma )$) with their values from the previous iteration. If the iterations converge, then they will give one of the possible solutions of the problem. Thus, the existence of the solution to the problem can be established *a-posteriori*. If the iterative process diverges, this will mean that there exists no solution to the problem.

### A.4 Iterative algorithm for the inverse sub-problem

Algorithm 1 shows the iterative procedure for solving the system (16)–(21), along with obtaining the values of the transmission and removal rates from equations (25) and (26).

### A.5 Validation of the numerical solution method

The accuracy of the developed numerical method and the corresponding algorithm are verified with mandatory tests involving different values of the time step $\tau$. We confirmed the practical convergence and the $O(\tau ^2)$ approximation of the difference scheme. We chose Israel for these tests because this country started mass vaccination of the population relatively early compared to other countries.

The computed numerical values of the identified coefficients $\beta$ and $\gamma$ with four different values of $\tau$ for a time period $P=28$ days for Israel are given in Table 2, from May 24, 2021 to June 21, 2021. The rates of convergence are computed using the formulas

27 $$\begin{aligned} \text {rate}_\beta = \log _2\frac{\beta _{\tau } - \beta _{2\tau } }{\beta _{2\tau } - \beta _{4\tau } }, \qquad \text {rate}_\gamma = \log _2\frac{\gamma _{\tau } - \gamma _{2\tau } }{\gamma _{2\tau } - \gamma _{4\tau } }. \end{aligned}$$

**Table 2:** Obtained values of the constants $\beta$ and $\gamma$ and the rate of convergence for four different values of the mesh spacing $\tau$.

$\tau$	$N\beta$	rate$_\beta$	$\gamma$	rate$_\gamma$
1/10	0.1005883672528720	–	0.0741311130168358	–
1/20	0.1005886480876050	–	0.0741311570649426	–
1/40	0.1005887182822550	2.0002884	0.0741311680719028	2.0006639
1/80	0.1005887358165650	2.0011804	0.0741311708184778	2.0027105
1/160	0.1005887402874910	1.9715351	0.0741311715365409	1.9354511

The tests confirm the second order of convergence of the numerical solution with respect to $\tau$. The results for other countries and time periods *P* are similar.
